# Necrotizing salpingitis in commercial laying hens associated with fowl adenovirus serotype 1 infection in China

**DOI:** 10.1016/j.psj.2026.107810

**Published:** 2026-09-21

**Authors:** Wei E, Hongxiang Liu, Can Zhang, Meili Wu, Ming Zou, Chenglong Zhao, Shanshan Song, Shaoyan Yang, Bin Li, Wei Jin, Weiwei Guo, Wenhua Liu, Shouzhen Xu, Yang Chen, Dongzu Luan, Zengzhi Qiu, Dong Liu

**Affiliations:** a Yebio Bioengineering Co., Ltd of Qingdao, Qingdao, 266100, China; b College of Veterinary Science, Qingdao Agricultural University, Qingdao, 266109, China; c Qingdao Laiyuanfa Biotechnology Co., Ltd., Qingdao, 266100, China

**Keywords:** Fowl adenovirus serotype 1, Necrotizing salpingitis, Egg drop syndrome, Epidemiological surveillance, Serological cross-reactivity

## Abstract

Fowl adenovirus serotype 1 (FAdV-A1) is a significant pathogen primarily associated with gizzard erosion and ulceration (GEU) in poultry. This study conducted a comprehensive epidemiological investigation of FAdV-A1 in 16,481 clinical samples collected from commercial poultry farms across 30 provinces in China between January 2022 and March 2026. The results revealed an overall FAdV-A1 detection rate of 0.36% (60/16,481), with 75% (45/60) of positive cases identified in laying hen flocks, predominantly during the onset to peak production phase (18-35 weeks of age). FAdV-A1 infection caused necrotizing salpingitis in affected hens, characterized by increased oviduct secretions, uterine edema, and necrosis and desquamation of glandular epithelial cells. Phylogenetic analysis based on the *hexon* gene demonstrated that all 19 sequenced FAdV-A1 isolates share 95.62-99.78% sequence identity with the apathogenic CELO strain. Serological analyses of AGP and VN revealed that antisera against currently circulating FAdV-C4 vaccine showed limited cross-neutralization against FAdV-A1 *in vitro*. FAdV-A1 challenge in laying hens induced characteristic necrotizing salpingitis lesions. This investigation demonstrates that FAdV-A1 is not restricted to causing GEU lesions but also elicits a previously unrecognized clinical manifestation of necrotizing salpingitis in Chinese laying hen flocks, thereby providing a scientific basis for accurate diagnosis, serological differentiation, and comprehensive prevention and control measures.

## Introduction

Fowl adenovirus (FAdV) infections in poultry have been extensively documented worldwide since the first description of the virus in 1949, with subsequent identification of associated diseases including inclusion body hepatitis (IBH) and hydropericardium-hepatitis syndrome (HHS) (El-Shall et al., 2022, Hess, 2000). FAdV is classified into five species (FAdV-A to FAdV-E), which are further differentiated into 12 serotypes (FAdV-1 to 7, FAdV-8a, FAdV-8b, FAdV-9 to 11) based on serum cross-neutralization reactions (Hess, 2000). FAdV-A1 is the sole serotype within species A and has been traditionally associated with gizzard erosion and ulceration (GEU) in young chickens (Abe et al., 2001, Lindgren et al., 2022). However, in 2022, Viviana Gonzalez-Astudillo and colleagues (Gonzalez-Astudillo et al., 2022) first reported that FAdV-A1 infection can cause necrotizing salpingitis in adult hens, resulting in oviduct damage similar to Egg Drop Syndrome (EDS), suggesting previously unrecognized pathogenic characteristics of this virus.

In recent years, FAdVs have been widely prevalent in Chinese poultry populations. Notably, although the detection rates of highly pathogenic serotypes (C4, D11, and E8a/E8b) have declined, those of non-vaccinated serotypes (such as A1, D2, and E7) have shown an increasing trend (Wang et al., 2018, Zhuang et al., 2023, Zhang et al., 2026). In November 2022, our laboratory first identified FAdV-A1 associated necrotizing salpingitis in a flock of 150-day-old laying hens in Yantai City, Shandong Province. Subsequently, similar cases were detected nationwide.

Currently, research on FAdV-A1-induced oviduct damage in chickens remains limited. In this study, large-scale epidemiological surveillance was performed from 2022 to March 2026, combined with pathogen isolation and identification, serological analysis, and experimental infection studies. The investigation will systematically elucidate the epidemiological characteristics of FAdV-A1 and its novel clinical manifestation of necrotizing salpingitis in Chinese chicken flocks, providing a scientific basis for the diagnosis and control of this disease.

## Materials and methods

### Viruses and reagents

FAdV-A1 strains SDYT-5328 and HeBTS-4183 were isolated from affected laying hens in laboratory of Yebio Bioengineering Co., Ltd of Qingdao. FAdV-C4 (strain BVA-4), FAdV-D11 (strain DQD2341), and FAdV-E8a (strain JSYC16809) were preserved at Yebio Bioengineering Co., Ltd of Qingdao. All virus stocks were propagated in LMH cells. PCR reagents were purchased from TaKaRa (Dalian, China). primers used in this study were synthesized by Beijing Tsingke Biotech Co., Ltd. (Beijing, China) (Table 1). SPF laying hens and Hy-Line Brown laying hens were purchased from Shandong Haotai Laboratory Animal Breeding Co., Ltd. (Shandong, China).

**Table 1:** Primers and amplification conditions.

Primers	sequences (5′—3′)	Tm	Length (bp)
FAdV-F	TTCCGCATGGCTGACGC	60°C	134
FAdV-R	CTTGGGGTCCGTTCTC		
FAdV-FAM	AGCGCCTGAAACCGAACAT		
FAdV-A1F	CGGATGACACGCAACAG	60°C	97
FAdV-A1R	TCGCCCCCATGTCCAA		
FAdV-A1-VIC	ATCGCGTGCGCTACAGCCAA		
FADV1384-F	GCCACCGGAAGCTACTTT	55°C	1384
FADV1384-R	AGGGTTGACGTTGTCCA		

Note: FADV1384 is the laboratory designation for the primer pair used in this study, where "1384" indicates the length (in base pairs) of the amplified PCR product fragment.

### Epidemiological investigation

#### Clinical sample collection and pathogen detection

A total of 16,481 clinical samples were collected from 7,347 commercial poultry farms across 30 provinces in China between January 2022 and March 2026. The samples were obtained from laying hens, broilers, and breeding chickens exhibiting various clinical symptoms, such as depression, diarrhea, respiratory symptoms, and egg production abnormalities. Sample types included carcasses, tissues, swabs, and dead embryos. Samples collected from each farm or hatchery were transported to the laboratory under cold chain conditions at 4°C, and the clinical information including flock age, breed, clinical signs, mortality rates, and production parameters was recorded for each case. Chickens and dead embryos were dissected for sampling, and the necropsy findings were documented.

Molecular identification and sequencing

Genomic DNA was extracted using commercial kits (Tiangen Biotech, Beijing) according to the manufacturer's instructions. Universal qPCR identification of FAdV was performed using specific primers FAdV-F/R/FAM designed based on the *hexon* gene. Positive samples were then subjected to Hexon gene amplification using primers FAdV1384-F/R. The PCR products were detected by 1% agarose gel electrophoresis and all positive products were sequenced by Shanghai Sangon Biotech Co., Ltd. (Shanghai, China). The sequencing results were blasted with the NCBI database to confirm FAdV-A1 positive samples. From 2025, FAdV-A1-specific qPCR using primers FAdV-A1F/R/VIC was also performed for enhancing detection sensitivity and specificity. Phylogenetic analysis of the FAdV-A1 sequences was conducted using the maximum-likelihood method implemented in IQ-TREE, with branch support evaluated by 1,000 bootstrap replicates. To exclude co-infections, all samples were additionally screened for avian influenza virus H9 (AIV H9), egg drop syndrome virus (EDSV), and Newcastle disease virus (NDV) by PCR; primer sequences and reaction conditions are listed in Table 1. The analytical sensitivity of each assay was determined using 10-fold serial dilutions of a quantified plasmid standard in triplicate, yielding LODs of 5 copies/μL for FAdV-F/R/FAM and FAdV-A1F/R/VIC (positivity threshold: Ct ≤ 35) and 300 copies/μL for FAdV1384-F/R. Assay specificity was confirmed by testing against a panel of common avian pathogens (AIV H9, EDSV, NDV, etc.), with no cross-reactivity detected.

### Virus isolation and inactivated vaccine preparation

#### Isolation of FAdV-A1

FAdV-A1-positive tissue samples were homogenized in PBS (pH 7.2), freeze-thawed three times, and centrifuged at 8,000 rpm for 10 min. Supernatants were filtered through 0.22 μm filters and inoculated onto LMH cells. Following 1 h of adsorption at 37**°C** with 5% CO_2_, the inoculum was removed and replaced with DMEM containing 2% fetal bovine serum (GIBCO). Cytopathic effect (CPE) was observed daily, virus was harvested after 2 passages. Virus titer was determined using the Reed-Muench method and shown as TCID_50_. Two FAdV-A1 strains (SDYT-5328 and HeBTS-4183) with TCID_50_ of 10^7.5^/0.1 mL and 10^7.83^/0.1 mL were propagated in LMH cells and inactivated with 0.2% formalin. The inactivated viruses were emulsified with Montanide ISA 71 VG adjuvant (SEPPIC, France) at a 3:7 ratio to prepare oil-adjuvanted inactivated vaccines.

#### Serological analysis

Twenty-four 53-day-old SPF chickens were randomly allocated to three groups (n=8 per group) and housed separately in isolators. Control group received no treatment. The other two groups were respectively inoculated with inactivated FAdV-A1 vaccines (strains SDYT-5328) and (HeBTS-4183) via intramuscular injection at 1 ml/chicken, with a booster immunization at 2 weeks post-primary vaccination. Serum samples were collected at pre-immunization (53 days), 28 days post-primary (81 days), and 42 days post-primary (95 days). The cross-reaction of the sera with FAdV-C4, FAdV-D11, and FAdV-E8a antigens was evaluated using the agar gel precipitation (AGP) test. Meanwhile, for cross-neutralization assays, antisera from chickens immunized with inactivated FAdV-A1 (HeBTS-4183) or FAdV-C4 vaccine were twofold serially diluted, mixed with 100 TCID₅₀ of FAdV-A1 (HeBTS-4183) at 37°C for 1 h, and then added to LMH cells. Neutralizing antibody titers were determined based on the presence of typical CPE. Standard positive serum (C4 vaccine strain) control, blank control, and virus controls (0.1, 1, 10, and 100 TCID_50_) were also included.

The geometric mean titer (GMT) and coefficient of variation (CV) of each group were calculated with the formulas as follows:

$$\log_{2}GMT=\frac{\sum_{n}^{i=1}k_{i}}{n}$$

$k_{i}$: the dilution index of the i-th sample

$$CV\left(\%\right)=\frac{S_{\log X}}{|\log\bar{10}X|}\times 100\%$$

*S*_log_*_X:_* the standard deviation of the common logarithm (log10)-transformed reciprocal antibody titers

#### Experimental infection

Twenty 210-day-old Hy-Line Brown laying hens, confirmed pathogen-free by qPCR (negative for FAdV, FAdV-A1, EDSV, AIV H9, IBV, and AEV) and seronegative for FAdV-A1 by virus neutralization test, were randomly divided into challenge and control groups (n = 10). The challenge group received 1 mL of undiluted HeBTS-4183 isolate (titer: 10^7.83^ TCID_50_/0.1 mL, equivalent to 10^8.83^ TCID_50_ per bird) via wing-vein injection; the control group received an equal volume of sterile saline. Clinical signs and mortality were recorded daily. Cloacal (CL) and oropharyngeal (OP) swabs were collected at 3, 5, 7, 10, and 14 d post-inoculation (dpi) for viral shedding quantification. At 14 dpi, surviving birds were euthanized. Gross lesions in the liver, proventriculus, spleen, ovary, magnum, uterus, kidney, cecal tonsil, and lung were scored on a 0–3 scale. Tissue samples were collected from these organs for histopathological analysis (H&E staining) and qPCR-based viral genome quantification. For blinded assessment, samples were fixed in 4% paraformaldehyde, re-coded, and submitted to Huayun (Shandong) Inspection and Quarantine Service Co., Ltd. for independent histopathological examination and diagnostic reporting. All procedures were approved by the Institutional Animal Care and Use Committee and complied with animal welfare regulations (IACUC2026070401).

#### Statistical analysis

Data were analyzed using GraphPad Prism 10. Antibody titers were log-transformed for calculation of GMT and CV. Group differences were analyzed using unpaired t-test. For the FAdV-A1 challenge experiment, intergroup differences in viral shedding from swabs, histopathological lesion scores, and viral loads in different tissues were analyzed by one-way ANOVA or the Mann-Whitney U test, shedding rates between swab types were compared using unpaired t-test, as appropriate, with *p* < 0.05 considered statistically significant.

## Results

### Epidemiological status of FAdV-A1

#### Detection of FAdV-A1

From January 2022 to March 2026, a total of 756 FAdV-positive samples were detected from 16,481 clinical samples using FAdV-specific primers, representing an overall detection rate of 4.59% (756/16,481). Of these, 280 samples were successfully amplified and sequenced, and 30 were identified as FAdV-A1. The remaining 476 samples failed to yield usable sequences due to low viral nucleic acid loads or poor amplicon quality. A total of 60 samples were confirmed as FAdV-A1 positive. Of these, 27 were identified by sequencing only, 30 by FAdV-A1-specific PCR only, and 3 by both methods. Among these sequencing-identified FAdV-positive samples,​ the proportion of FAdV-A1 varied annually: 10.64% (5/47) in 2022, 9.46% (7/74) in 2023, 13.95% (12/86) in 2024, 5.00% (3/60) in 2025, and 23.08% (3/13) in 2026 (Table 2).

**Table 2:** Detection of FAdV and FAdV-A1 in different years.

Year	collected samples		FAdV-positive samples		FAdV-A1-positive samples	
	**farms**	**samples**	FADV-specific PCR	Sequencing-confirmed	A1-specific PCR	Sequencing- confirmed
2022	1,193	2,332	69	47	-	5
2023	1,695	3,335	116	74	-	7
2024	1,649	3,657	115	86	-	12
2025	2,183	5,534	356	60	29	3
2026 (-Mar)	627	1,623	100	13	4	3
Total	7,347	16,481	756	280	33	30

#### Geographical and flock distribution

The 60 FAdV-A1-positive samples were distributed across 10 provinces, with Shandong Province accounting for 43.33% of cases (Fig. 1). These cases involved various poultry types: laying hens (0.81%), yellow feather broiler (0.15%), white feather broiler (0.16%), broiler breeders (0.06%), and other types including embryos and environmental samples (0.76%). Laying hen flocks had the highest detection rate, with 45 positive samples detected from 35 layer farms (74.47%, 35/47) (Table 3). Notably, FAdV-A1 positive samples were detected from both embryos and their respective breeder flocks, suggesting a possibility of vertical transmission. The 6 white-feather-broiler flocks containing 9 FAdV-A1-positive samples exhibited high mortality owing to co-infection with IBV, AIV H9 and IBDV, making it impossible to determine clinical signs induced by FAdV-A1 alone. In the 35 FAdV-A1-positive laying-hen flocks, NDV, EDSV and AIV H9 were not detected. In addition, based on clinical symptoms, these flocks were selectively tested for other pathogens, including Tembusu virus (TMUV), Avian Encephalomyelitis Virus (AEV), Infectious Bursal Disease Virus (IBDV), Avian Leukosis Virus (ALV), Chicken Anemia Virus (CAV), Chicken hepatitis E virus (HEV), Marek's Disease Virus (MDV), Infectious Bronchitis Virus (IBV), Mycoplasma synoviae (MS), Mycoplasma gallisepticum (MG), Riemerella anatipestifer (RA), Infectious Coryza, Avibacterium paragallinarum (IC), Reticuloendotheliosis Virus (REV), Avian Reovirus (ARV), and Infectious Laryngotracheitis Virus (ILTV), and almost no co-infections with these pathogens were detected.

**Fig. 1:**
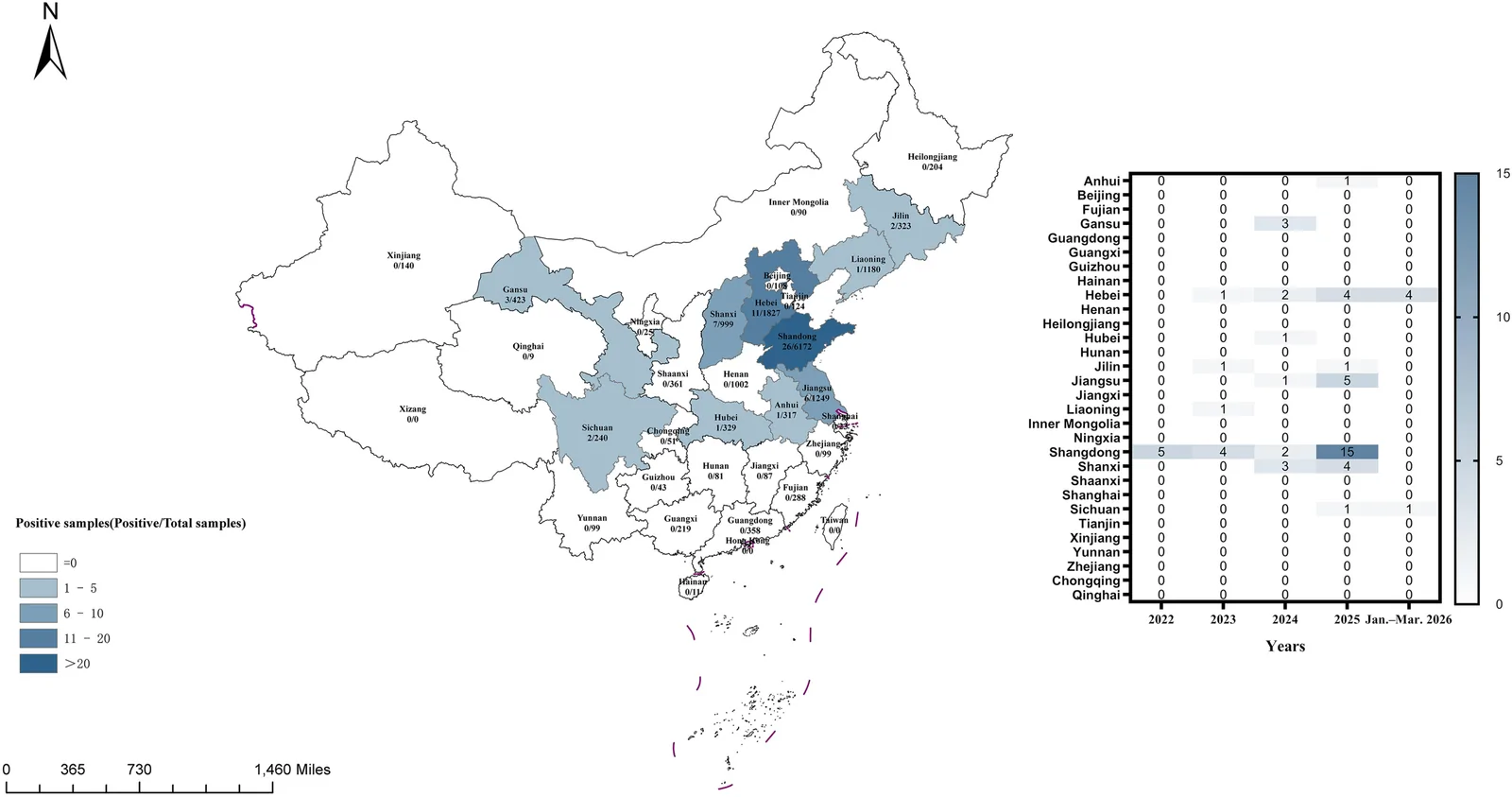
Provincial geographical distribution of FAdV-A1-positive samples in China from January 2022 to March 2026.

**Table 3:** Detection of FAdV-A1 in different poultry populations.

Population	Collected samples		FAdV-A1 **positive**	
	**Farms**	**Samples**	**FAdV-A1-positive farms**	**FAdV-A1-positive samples**
Laying hens	3,196	5,556	35	45
Broiler breeders	1,120	3,567	2	2
White-feather broilers	2,268	5,741	6	9
Yellow-feather chickens	697	1,354	2	2
Others (embryos, environmental swabs, etc.)	66	263	2	2
**Total**	7,347	16,481	**47**	**60**

#### Clinical signs and pathological changes

Given the low detection rate of FAdV-A1 in broiler breeders and embryo, and the fact that white feather broiler flocks had co-infections with other pathogens (IBV, AIV H9, and IBDV), clinical features and pathology were analyzed only on the 35 positive layer farms. The results are shown in Table 4. Among 35 positive laying hen flocks, most infections (24/35) occurred during onset to peak production (18-35 weeks of age). Most affected flocks showed no obvious clinical signs; 22/35 flocks had slightly increased mortality/culling, 10/35 had a minor decrease in egg production, and 9/35 had poor egg quality. Necropsy findings included oviduct secretions (20/35), uterine edema/mucus, yolk peritonitis (16/35), proventricular hemorrhage (15/35), follicle deformities (13/35), liver swelling (12/35) (Fig. 2) and rare kidney lesions (4/35). Uterine histopathology showed glandular epithelial necrosis and shedding (Fig. 3).

**Table 4:** Age distribution and clinical signs of FAdV-A1-positive laying hen flocks.

Age	Positive farms	Mortality impact			Egg production impact			Pathological changes					
		Severe (≥0.3%)	Slight (0.01% to <0.3%)	Little/No (<0.01%)	Production decline (>1%)	Eggshell quality (>0.5% unqualified)	Minor/no impact	Proventricular hemorrhage	Liver swelling	Oviduct secretions	Follicle deformation	Yolk peritonitis	Kidney swelling
0-3W	1	-	-	1	/	/	/	-	-	-	-	-	-
4-6W	-	-	-	-	/	/	/	-	-	-	-	-	-
7-9W	-	-	-	-	/	/	/	-	-	-	-	-	-
10-12W	1	-	1	-	/	/	/	-	-	-	-	-	1
13-18W	3	-	2	1	-	-	-	-	2	-	-	-	1
19-24W	10	-	6	4	3	5	4	3	1	4	3	5	-
25-35W	14	-	9	5	4	3	7	8	6	11	6	9	2
>35W	6	-	4	2	3	1	3	4	3	5	4	2	-

**Fig. 2:**
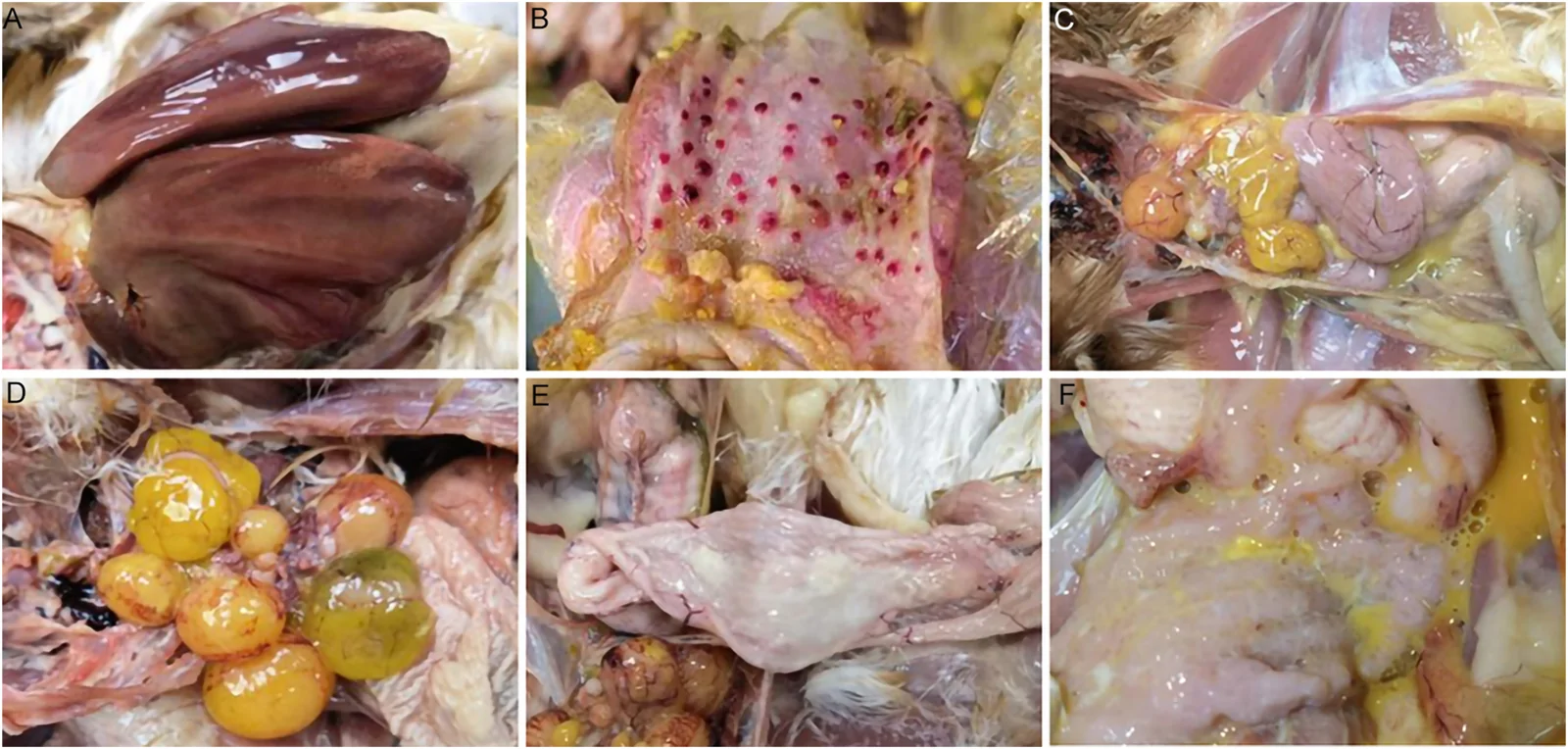
Clinical lesions and pathological changes in FAdV-A1-positive laying hens during the peak production period. (A) Liver swelling; (B) Proventricular hemorrhage; (C) Yolk peritonitis; (D) Follicular deformation and necrosis; (E) Whitish secretions in the oviduct (magnum); (F) Uterine edema with yolk-colored secretions.

**Fig. 3:**
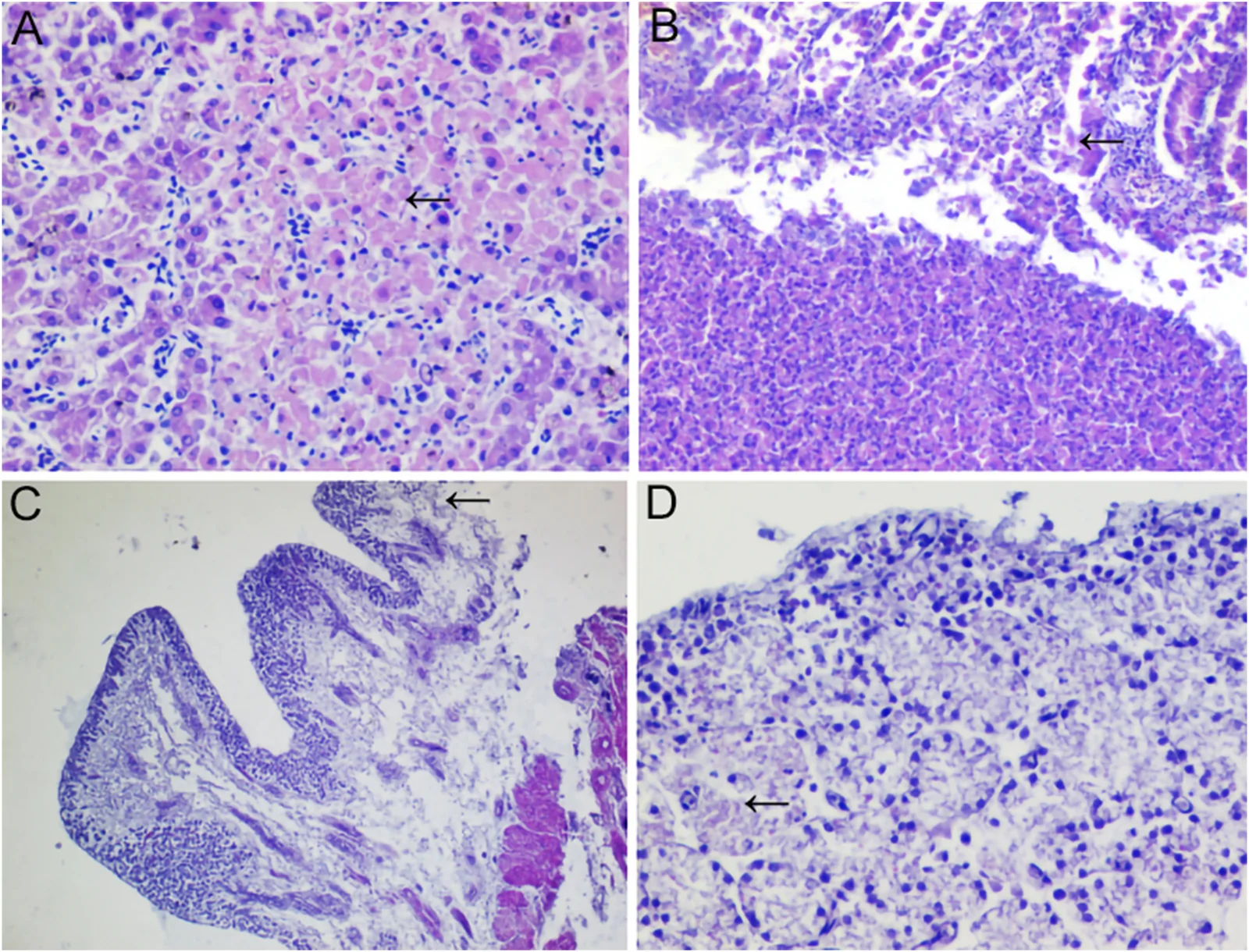
Histopathological changes in FAdV-A1-positive laying hens during the peak production period. (A) Focal necrosis of hepatocytes with only basophilic-staining nuclei remaining (histopathological change); HE×400. (B) Necrosis and desquamation of glandular epithelial cells in the proventricular submucosal glands forming epithelial necrosis foci; HE×200. (C) Necrosis and disintegration of glandular epithelial cells in the longitudinal folds of the oviduct (magnum); HE×100. (D) Necrosis and desquamation of mucosal epithelial cells and degeneration and necrosis of glandular epithelial cells in the longitudinal folds of the oviduct (uterus); HE×200.

#### Phylogenetic analysis

Nineteen FAdV-A1 isolates were selected for sequencing and phylogenetic analysis based on *hexon* gene (Table S2). All isolates clustered within the FAdV-A1 clade and were clearly separated from FAdV-B, -C, -D, and -E reference strains (100% bootstrap at the serotype level; Fig. 4). Notably, 12 of the 19 isolates formed a distinct, moderately supported sub-clade (76.1% bootstrap) sister to the reported A1 strains, suggesting a China-localized lineage. Sequence identity with the apathogenic CELO strain (NC_001720.1) ranged from 95.62% to 99.78%.

**Fig. 4:**
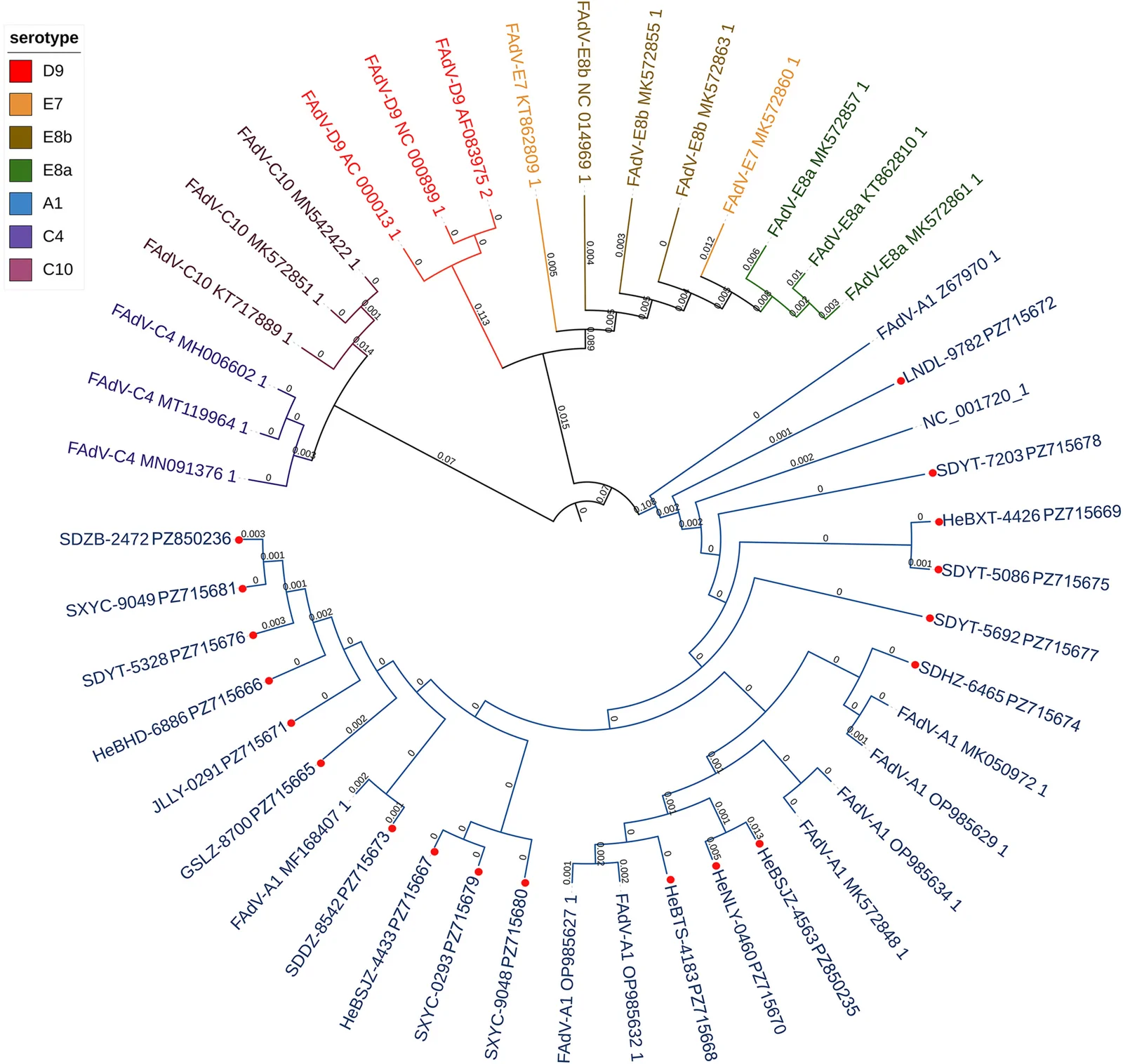
Phylogenetic tree based on the *hexon* gene of adenoviruses (FAdVs). Branch colors represent different serotypes, and red dots indicate isolation strains in this study.

#### Serological analysis

SPF chickens were immunized with inactivated vaccines prepared from the clinical isolates SDYT-5328 and HeBTS-4183 to produce antisera. The sera antibody titers were 1:2^3.13^ (CV=43.40%) and 1:2^4.00^ (CV=35.36%), respectively (Fig. 5A). AGP cross-reactivity assay showed that over 70% of post-booster sera reacted positively with FAdV-C4, FAdV-D11, and FAdV-E8a antigens, while control sera were all negative, indicating cross-reactivity between FAdV-A1 and other serotypes. VN tests showed that antisera collected after the second immunization with HeBTS-4183 had a VN titer of 1:2^6.63^ (CV=29.02%), whereas the VN titer of FAdV-C4 vaccine antiserum against HeBTS-4183 was only 1:2^0.75^ (CV = 61.72%), indicating lower heterologous neutralizing efficacy (Fig. 5B).

**Fig. 5:**
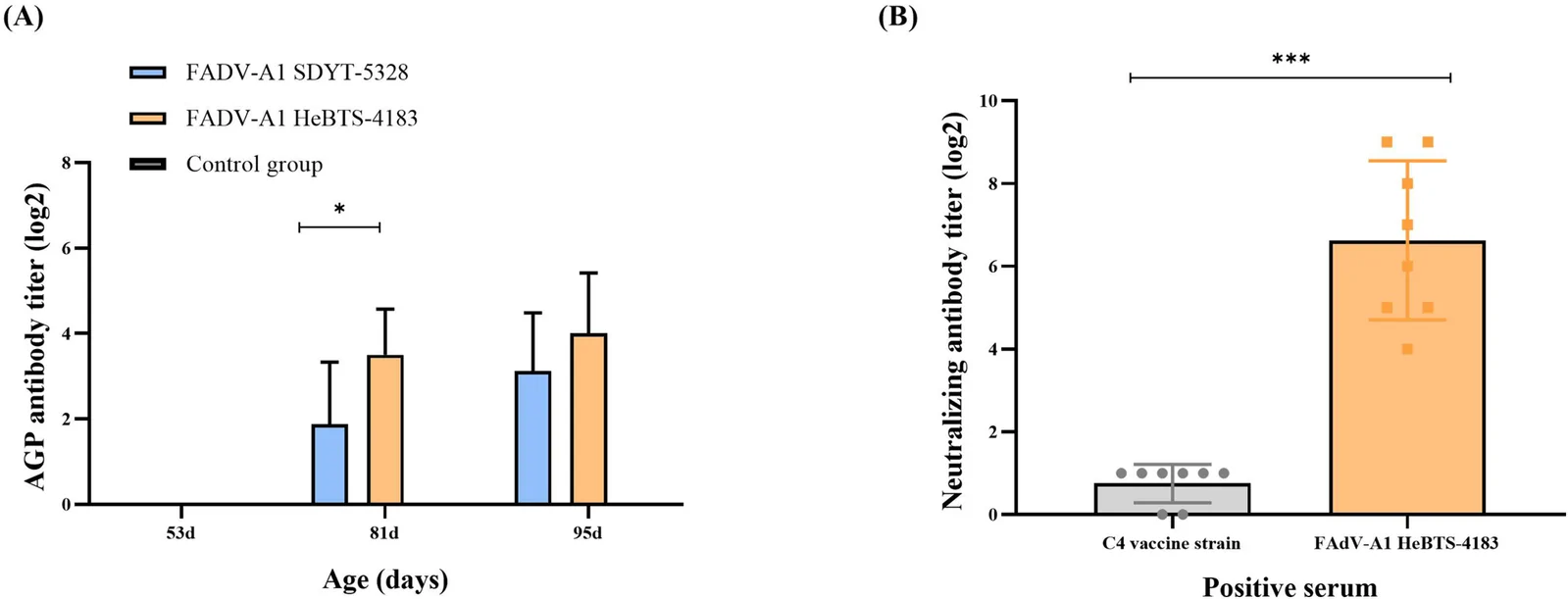
Antibody responses in chickens immunized twice with FAdV-A1 inactivated vaccines. (A) AGP antibody titers post-vaccination (mean ± SD; unpaired t-test; *p < 0.05). (B) Cross-neutralizing VN titers between HeBTS-4183 and FAdV-C4 strains (mean ± SD; unpaired t-test; ***p < 0.0001).

#### Experimental infection

All challenged birds received 1 mL of undiluted HeBTS-4183 via wing-vein injection and were observed over 14 d. No mortality was observed, and all birds remained clinically healthy except for a mild reduction in feed intake. CL and OP swabs were collected at 3, 5, 7, 10, and 14 dpi for qPCR analysis. Viral shedding was consistently low (Ct > 25), with a peak at 5 dpi (90% positivity) and a gradual decline thereafter; at 14 dpi only 40% of birds were positive in either swab type. Shedding rates did not differ significantly between swab types (t-test, *p* > 0.05; Fig. 6A).

**Fig. 6:**
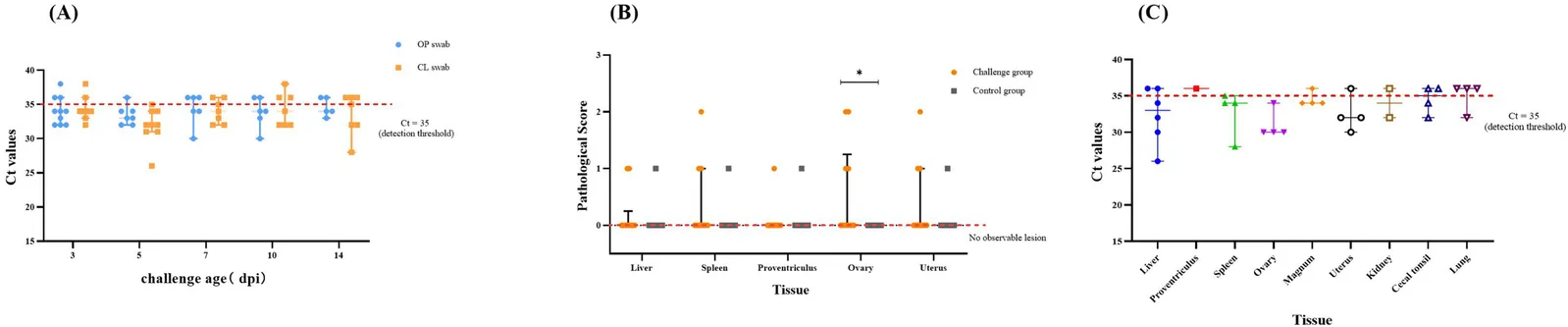
(A) Viral shedding in OP and CL swabs. (B) Tissue-specific pathological scores in challenge (orange) vs. control (gray) groups. Lesions were scored on a 0–3 scale (0 = no lesion). Data are shown as Medium with IQR. (C) Tissue distribution of FAdV-A1 viral load in the challenge group.

At 14 dpi, all birds were euthanized and necropsied. Gross lesions were scored 0–3 for each organ. Most challenged birds showed no obvious gross abnormalities, whereas 4/10 exhibited marked uterine edema (Fig. 7a), follicular necrosis (Fig. 7b), mild magnum congestion (Fig. 7c), and splenic enlargement (Fig. 7d). Histopathological examination revealed necrosis, congestion, and edema of uterine fold glands (Fig. 8a), interstitial congestion in the magnum (Fig. 8b), congestion and hemorrhage in the splenic red pulp (Fig. 8c), and congestion and hemorrhage of the follicular membrane (Fig. 8d). No or only mild lesions were observed in other organs or in the control group. Intergroup comparison of lesion scores revealed a significant difference only in the ovary (Mann-Whitney U test, *p* < 0.05; Fig. 6B).

**Fig. 7:**
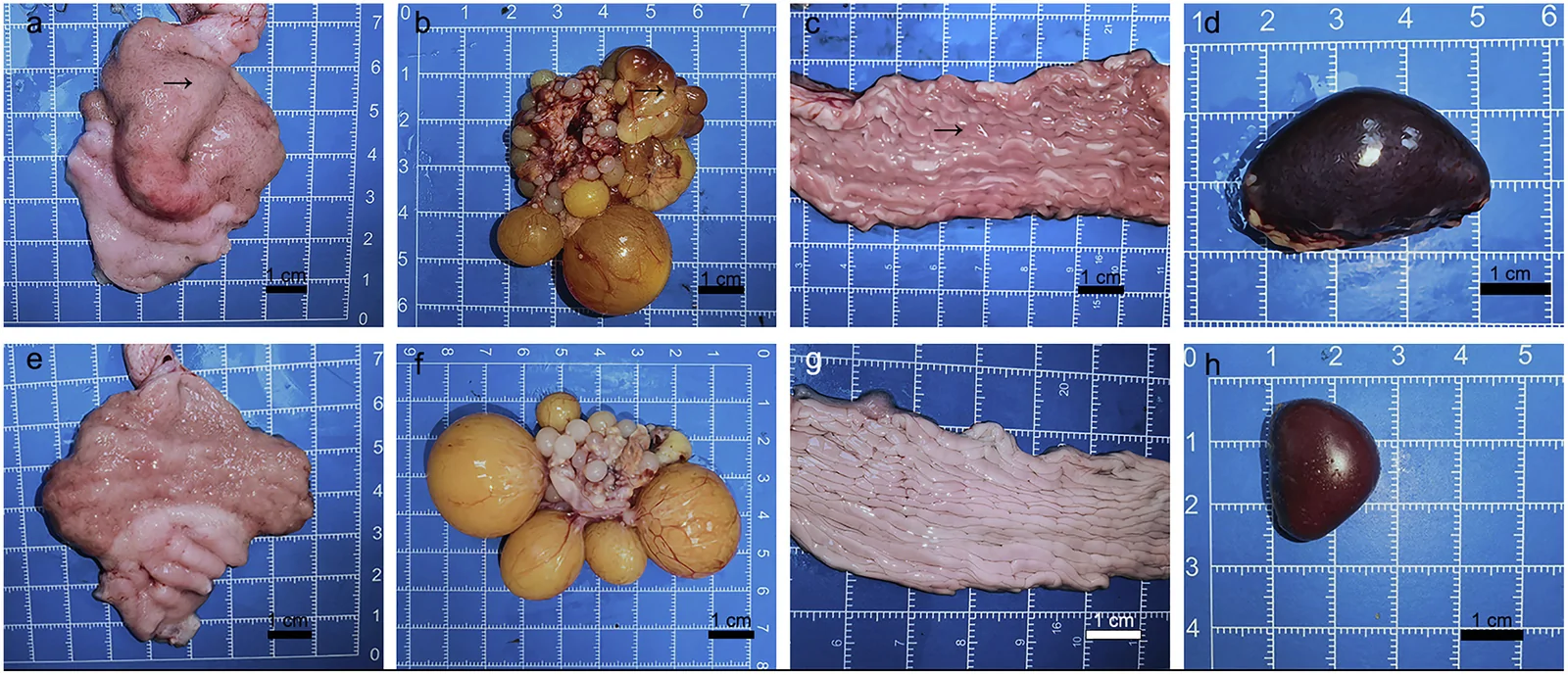
Gross pathological findings in each experimental group. Challenge group: (a) uterus edema with a small amount of transparent mucus; (b) deformed, liquefied, and necrotic ovarian follicles; (c) mild congestion of the magnum with a small amount of transparent mucus in the lumen; (d) splenic enlargement with a dark red appearance. The corresponding tissues in the control group (e: uterus; f: ovary; g: magnum; h: spleen) showed no obvious lesions or only mild changes.

**Fig. 8:**
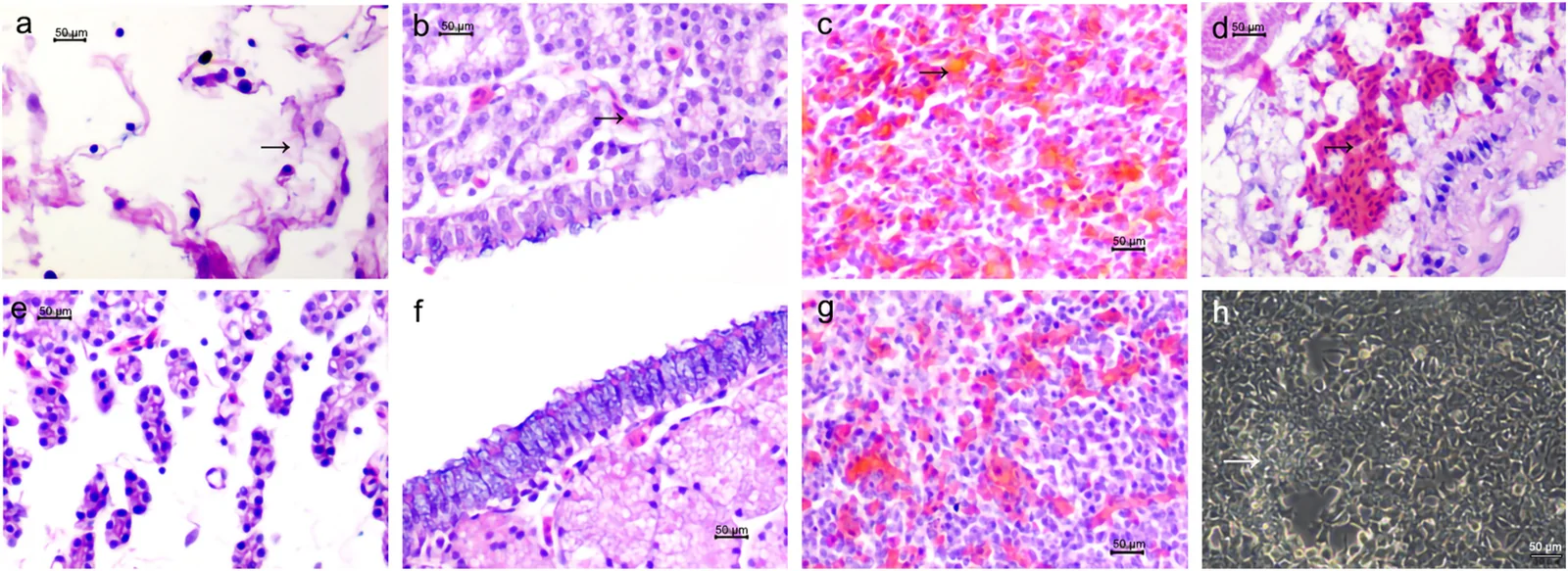
Histopathological changes (H&E staining, ×400) and CPE in LMH cells (a-h). Challenge group: (a) intact epithelial cell structure of the uterine fold mucosa, with necrosis, congestion, and edema of the fold glands; (b) vacuolar degeneration of resting glandular epithelial cells in the magnum, with interstitial congestion; (c) congestion and hemorrhage in the splenic red pulp; (d) homogeneous eosinophilic droplet-like necrotic yolk material, with congestion and hemorrhage in the follicular membrane. The corresponding tissues in the control group (e: uterus; f: magnum; g: spleen) showed no obvious lesions or only mild changes. (h) CPE in LMH cells at 72 h post-infection with the F2-generation virus recovered from tissue homogenates (×200). Cells became round, detached, and exhibited enhanced refractility (arrows), consistent with characteristic FAdV-induced CPE.

Various organs were collected from each group for qPCR detection. In the challenge group, no significant differences in Ct values were observed among organs (ANOVA, *p* > 0.05; Fig. 6C). The highest detection rates were observed in liver (40%), ovary (40%), and spleen (40%), followed by uterus (30%), while lower rates were found in magnum (20%), cecal tonsil (20%), kidney (10%), and lung (10%); no viral DNA was detected in the proventriculus (0%). Tissue samples with Ct values ≤ 30 (n = 7) were processed and inoculated onto LMH cells for virus isolation. After blind passage, two samples (liver and uterus) exhibited obvious CPE at the second passage, were confirmed as FAdV-A1-positive by qPCR and Sanger sequencing.

## Discussion

Since the first isolation of FAdV in 1949, the virus has been detected in various avian species (Van Den Ende et al., 1949). In 1963, the first case of FAdV-associated IBH in the United States was reported (Helmboldt and Frazier, 1963), and subsequently, the virus was isolated from chicken flocks with IBH or hydropericardium-hepatitis syndrome (HHS) worldwide (El-Shall et al., 2022). FAdV-A1 is the sole serotype within species A. Kiss et al. (2021) reported that FAdV-A1 accounted for 11% of 365 isolates from 38 countries between 2008-2019. In South Korea, FAdV-A1 was only detected in 2010, 2018 and 2021 (2.2%, 4.8% and 2.1% respectively) (Mo, 2021, Kim et al., 2025).

In China, FAdVs have been prevalent in commercial poultry during 2007-2017, with dominant serotypes C4, D11, and E8a/E8b associated with IBH/HHS (Wang et al., 2018). Following the large-scale FAdV outbreaks caused by C4/D11/E8a during 2013–2017, serotype-matched vaccines were progressively deployed, after which the detection rates of these serotypes declined, while non-vaccinated serotypes (A1, D2, E7) have increased. Zhuang et al. (2023) detected FAdV in 166 positive samples (detection rate 8.65%) from healthy commercial poultry in eastern China, with FAdV-A1 accounting for 18.07%. Zhang et al. (2026) reported FAdV prevalence of 2.42% in 18,297 samples collected from 29 provinces between 2021-2025, with FAdV-A1 accounting for 12.80%. Consistent with previous reports, the present study showed that the sample-level FAdV detection rate ranged from 2.94% to 6.43%. Among 280 FAdV-positive sequenced samples derived from 7,347 commercial poultry flocks during 2022–2026, FAdV-A1 accounted for 10.71% (30/280).

Unlike serotypes C4, D11, and E8b, which mainly cause IBH and HHS, FAdV-A1 is more frequently associated with GEU in chickens. Abe et al. (2001) first observed AGE symptoms associated with FAdV-A1 in 17-day-old broilers, characterized by erosion of the koilin layer. Subsequent reports confirmed this association (Kardoudi et al., 2025, Montagnini et al., 2026). Commercial broiler flocks are susceptible, mainly presenting with poor growth. In this study, only 6 out of 2,268 white feather broiler flocks tested positive for FAdV-A1, and were found to be co-infected with other pathogens (IBV, AIV H9, IBDV, etc.), presenting complex clinical signs without typical AGE lesions. Matczuk et al. (2017) first reported that FAdV-A1 could reduce egg production in adult laying hens. They attributed the production decline to indirect effects of gizzard erosion rather than direct viral tropism for the reproductive tract. Gonzalez-Astudillo et al. (2022) observed necrotizing salpingitis in a 3-year-old backyard hen, which was the first single case report of FAdV-A1 associated oviduct lesions. In November 2022, our laboratory first identified FAdV-A1 in 150-day-old commercial layers associated with EDS-like symptoms. Necropsy revealed ovarian atrophy, proventriculitis, oviduct distension with white exudate, and uterine edema, with FAdV-A1 as the sole detected pathogen. Subsequently, flock-level prevalence and production data were collected. A total of 35 A1-positive laying hen flocks were identified from 3,196 layer flocks, of which 30/35 were in production phase (>126 days old), mostly at onset or peak production (80%, 24/30). The concentration of positive cases in laying hens during peak production raises the hypothesis that reproductive stress may facilitate viral pathogenesis, and further studies are needed to confirm this association. Our study provides the first large-scale epidemiological evidence that FAdV-A1-associated salpingitis is not an isolated incident but a recurring problem in commercial layer flocks, thereby establishing the clinical and economic significance of this infection during the production period.

Of note, FAdV-A1 can also be detected in healthy laying hen flocks. Jakab et al. (2023) reported FAdV-A1 positivity in healthy flocks worldwide, and Zhuang et al. (2023) identified A1 as a dominant serotype in healthy commercial poultry in China. In this study, FAdV-A1 was also detected in healthy broiler breeders, broilers, and laying hens without clinical signs. For example, the same laying hen flock tested positive for FAdV-A1 three times (150-180 days of age) while remaining clinically normal. The detection of FAdV-A1 in clinically healthy flocks and embryos suggests potential vertical transmission and warrants further investigation into its epidemiological dynamics. Notably, co-infections were not identified among FAdV-A1-positive samples, supporting the possibility that FAdV-A1 can act as an independent primary pathogen causing reproductive lesions in commercial laying hens under field conditions.

To verify the pathogenicity of the field-derived HeBTS-4183 strain (FAdV-A1), a challenge trial was conducted using 210-day-old Hy-Line Brown laying hens, mirroring the age and production stage of the original affected flock. Infected chickens exhibited persistent low-level viral shedding, with only 40% of birds still shedding virus by 14 dpi. At necropsy, while most challenged birds showed no obvious gross abnormalities, 4/10 hens displayed marked uterine edema, follicular necrosis, and splenic enlargement. FAdV-A1 was successfully re-isolated from one liver and one uterus sample among seven tissues with Ct values ≤ 30, confirming active viral replication. The inconsistent development of uterine lesions (4/10 birds) and the limited virus re-isolation (2/7 tissues) likely reflect the low virulence of FAdV-A1, individual host variation in viral load and reproductive maturity, and the decline in infectious virus by 14 dpi. Together, these factors may have permitted persistent detection of viral DNA while hindering the recovery of live virus. Notably, despite this relatively high hepatic viral load, neither gross nor histopathological liver lesions were observed. This is consistent with field reports that FAdV-A1 in adult birds causes salpingitis rather than IBH and parallels the mild production impacts seen in most affected field flocks, which align with previous descriptions of subclinical FAdV-A1 infections (Kardoudi et al., 2025). Together, the tissue-specific lesion distribution and absence of hepatic pathology indicate that the HeBTS-4183 strain possesses relatively low virulence, with tropism restricted primarily to the reproductive tract. The characteristic necrotizing salpingitis observed in both field cases and experimental infection suggests that FAdV-A1 targets the oviduct, potentially impairing egg quality through glandular epithelial damage.

The FAdV-A1 genome was highly conserved within serotype. Genetic variation occurs primarily through point mutations and insertions/deletions, concentrated in hypervariable regions with minimal changes in hexon and fiber genes (Matczuk et al., 2017, Raue and Hess, 1998). The hexon is a key target for serotyping and differentiation (Raue and Hess, 1998). In this study, the phylogenetic analysis of 19 FAdV-A1 isolates confirms close relatedness to known FAdV-A1 strains, including the apathogenic CELO strain. Thanasut et al. (2017) found AGE clinical isolates had 99% homology with the CELO strain, but with insertions/deletions in multiple ORFs and the L4 and L5 regions. Del Valle et al. (2020) isolated two non-virulent C4 strains from the oviducts of laying hen flocks with decreased egg production. This suggests virulence may be strain-specific or context-dependent (Jakab et al., 2023).

AGP is widely used in China for FAdV antibody detection and clinical diagnosis, but cannot differentiate serotypes. VN is the gold standard for differentiating all 12 serotypes due to its high sensitivity and specificity (Grimes et al., 1977), requiring ≥16-fold difference between homologous and heterologous titers (Hess, 2000). In this study, serological assay showed a high cross-reactivity with C4, D11, and E8a in AGP but a poor cross‑neutralization between FAdV-C4 and FAdV-A1 in VN, indicating limited cross-neutralizing activity *in vitro*. Notably, all FAdV-A1-positive flocks had been vaccinated with commercial FAdV vaccines, yet they remained susceptible to A1 infection under field conditions. Although *in vitro* VN test cannot replace *in vivo* challenge studies, the actual protective efficacy of current commercial vaccines against FAdV-A1 requires further evaluation through active immunization-challenge experiments. The non-pathogenic CELO strain may be used as a vaccine candidate for the prevention of FAdV-A1 (Grafl et al., 2014, Grafl et al., 2020), but its protective efficacy remains to be validated in future studies.

## Conclusion

This study provides the first comprehensive evidence that FAdV-A1 is an emerging cause of necrotizing salpingitis in commercial laying hen flocks in China. FAdV-A1 was detected in 0.36% (60/16,481) of samples across 30 provinces, with 75% of positive cases concentrated in laying hen flocks, predominantly during the onset to peak production period (18-35 w). The disease is characterized by necrosis and desquamation of glandular epithelial cells in the oviduct, leading to mild declines in egg production and shell quality. Notably, current vaccines targeting FAdV-C4 elicit antibodies with limited *in vitro* cross-neutralization against FAdV-A1. These findings underscore the need for better diagnostics and A1-targeted prevention, advance understanding of FAdV-A1 pathogenesis, and inform control of this overlooked poultry pathogen.

## Author contributions

All authors made substantial contributions to the study's design, data acquisition, analysis, or interpretation; drafted or critically revised the manuscript; approved the final version; and agreed to be accountable for all aspects of the work.

**Wei E:** Conceived and designed the study, carried out the PCR and serological assays, field investigation, and data analysis, and drafted the manuscript. **Hongxiang Liu:** Conceived and designed the study, carried out the PCR and serological assays, field investigation, and data analysis, and drafted the manuscript. **Can Zhang:** Performed genetic sequence analysis and data processing, participated in data interpretation, and critically revised the manuscript. **Meili Wu:** Participated in virus strain isolation and animal experiments, participated in data interpretation, and critically revised the manuscript. **Ming Zou:** Performed data processing and statistical analysis, participated in data interpretation, and critically revised the manuscript. **Chenglong Zhao:** Participated in sample processing and laboratory testing, participated in data interpretation, and critically revised the manuscript. **Shanshan Song:** Participated in sample processing and laboratory testing, participated in data interpretation, and critically revised the manuscript. **Shaoyan Yang:** Collected clinical information and samples, participated in data interpretation, and critically revised the manuscript. **Bin Li:** Collected clinical information and samples, participated in data interpretation, and critically revised the manuscript. **Wei Jin:** Collected clinical information and samples, participated in data interpretation, and critically revised the manuscript. **Weiwei Guo**: Participated in study conceptualization, provided project supervision and resources, and critically revised the manuscript. **Wenhua Liu:** Participated in sample processing and laboratory testing, participated in data interpretation, and critically revised the manuscript. **Shouzhen Xu:** Participated in sample processing and laboratory testing, participated in data interpretation, and critically revised the manuscript. **Yang Chen:** Participated in virus strain isolation and animal experiments, participated in data interpretation, and critically revised the manuscript. **Dongzu Luan:** Collected clinical information and samples, participated in data interpretation, and critically revised the manuscript. **Zengzhi Qiu:** Collected clinical information and samples, participated in data interpretation, and critically revised the manuscript. **Dong Liu:** Conceived and designed the study, provided overall project supervision, funding acquisition, and resources, and critically revised the manuscript.

All authors approved the final manuscript and agreed to be accountable for the accuracy and integrity of the work.

## Ethics declaration

This study was conducted in accordance with the ARRIVE (Animal Research: Reporting of In Vivo Experiments) guidelines. This study was approved by the Institutional Animal Care and Use Committee of Yebio Bioengineering Co., Ltd of Qingdao. (Approval No. IACUC2026020501 and IACUC2026070401)

## Disclosures

All authors read and approved the final manuscript; no conflict of interest exists in the submission of this manuscript.

## Footnotes

Supplementary material associated with this article can be found, in the online version, at doi:10.1016/j.psj.2026.107810.
